# Cancer Stem Cells: A Minor Cancer Subpopulation that Redefines Global Cancer Features

**DOI:** 10.3389/fonc.2013.00076

**Published:** 2013-04-15

**Authors:** Heiko Enderling, Lynn Hlatky, Philip Hahnfeldt

**Affiliations:** ^1^Center of Cancer Systems Biology, St. Elizabeth’s Medical Center, Tufts University School of MedicineBoston, MA, USA

**Keywords:** mathematical model, cellular automaton, cancer stem cell, symmetric division, invasion, morphology

## Abstract

In recent years cancer stem cells (CSCs) have been hypothesized to comprise only a minor subpopulation in solid tumors that drives tumor initiation, progression, and metastasis; the so-called “cancer stem cell hypothesis.” While a seemingly trivial statement about numbers, much is put at stake. If true, the conclusions of many studies of cancer cell populations could be challenged, as the bulk assay methods upon which they depend have, by, and large, taken for granted the notion that a “typical” cell of the population possesses the attributes of a cell capable of perpetuating the cancer, i.e., a CSC. In support of the CSC hypothesis, populations enriched for so-called “tumor-initiating” cells have demonstrated a corresponding increase in tumorigenicity as measured by dilution assay, although estimates have varied widely as to what the fractional contribution of tumor-initiating cells is in any given population. Some have taken this variability to suggest the CSC fraction may be nearly 100% after all, countering the CSC hypothesis, and that there are simply assay-dependent error rates in our ability to “reconfirm” CSC status at the cell level. To explore this controversy more quantitatively, we developed a simple cellular automaton model of CSC-driven tumor growth dynamics. Assuming CSC and non-stem cancer cells (CC) subpopulations coexist to some degree, we evaluated the impact of an environmentally dependent CSC symmetric division probability and a CC proliferation capacity on tumor progression and morphology. Our model predicts, as expected, that the frequency of CSC divisions that are symmetric highly influences the frequency of CSCs in the population, but goes on to predict the two frequencies can be widely divergent, and that spatial constraints will tend to increase the CSC fraction over time. Further, tumor progression times show a marked dependence on both the frequency of CSC divisions that are symmetric and on the proliferation capacities of CC. Together, these findings can explain, within the CSC hypothesis, the widely varying measures of stem cell fractions observed. In particular, although the CSC fraction is influenced by the (environmentally modifiable) CSC symmetric division probability, with the former converging to unity as the latter nears 100%, the CSC fraction becomes quite small even for symmetric division probabilities modestly lower than 100%. In the latter case, the tumor exhibits a clustered morphology and the CSC fraction steadily increases with time; more so on both counts when the death rate of CCs is higher. Such variations in CSC fraction and morphology are not only consistent with the CSC hypothesis, but lend support to it as one expected byproduct of the dynamical interactions that are predicted to take place among a relatively small CSC population, its CC counterpart, and the host compartment over time.

## Introduction

Normal tissues undergo constant turnover, with cells dying due to age, injury, or shedding, and being replaced by new healthy cells. Homeostasis is accomplished by a potent subpopulation of stem cells. In recent years, a potent subpopulation of stemlike cells has also been proposed to exist as a minority population in cancers. First in leukemia and later in solid tumors, distinct cell populations were isolated that were either capable or not capable of initiating and sustaining, and re-initiating tumor growth (Furth and Kahn, 1937; Al-Hajj et al., 2003; Singh et al., 2003; Visvader and Lindeman, 2008; Vlashi et al., 2009). The picture to emerge – that of a potent cancer stem cell (CSC) that initiates and progresses the tumor, with the bulk of the growing tumor being composed of replication-limited cancer cells (CCs) – stands in marked contrast to the long-established paradigm that cancer cells typically are long-lived, escape cell death, and have limitless replicative potential (Hanahan and Weinberg, 2000, 2011), and argues against one recent study (Quintana et al., 2008), which has been interpreted to suggest we may simply be “under-assaying” the preponderant stem cell population (Baker, 2008). Summing up this alternative paradigm is the CSC hypothesis, perhaps better described as a cancer “non-stem cell” hypothesis, which posits that in fact only a few cells in the tumor population exhibit the immortal, stemlike trait. Confusion sometimes accompanying the CSC terminology regards the cell of origin of the disease. While the term “CSC” clearly suggests these cells possess stemlike qualities, this should not be taken to suggest they originate from normal stem cells. Indeed, the literature is split on the matter of origin (Haeno et al., 2009; Rahman et al., 2009; Shibata and Shen, 2013), with debates occurring occasionally even within the same tumor type, as in the case in glioblastoma (Stiles and Rowitch, 2008; Hambardzumyan et al., 2011).

Populations enriched for stemness can be isolated using different surface markers. The number of cells from such enriched populations that is necessary to form tumors gives an indication of the fraction of cells that are CSCs in the primary tumor. We calculated these ratios from data reported in the literature (Visvader and Lindeman, 2008) (Table 1), and found these tend to support numerous other reports that CSCs are indeed a rare population within a tumor (Reya et al., 2001; Pardal et al., 2003). In addition to identification of CSCs through surface proteins *in vitro* and *in vivo* mouse xenograft transplantation assays, novel approaches emerge that trace tumor hierarchy and help estimate CSC kinetics and frequency in spontaneous tumors or orthotropic models. One approach to monitor the division kinetics of stem and progenitor cells in normal epithelial tissues, skin papilloma, and invasive squamous cell carcinoma during unperturbed growth emerged from clonal analysis using genetic lineage tracing in mice (Driessens et al., 2012). Gao et al. (2013) used an integrated experimental and cellular Potts model approach to simulate glioblastoma population growth and response to irradiation, which identified the (a)symmetric division kinetics of glioblastoma stem cells necessary to reproduce the observed ratio of 2–3% of such cells. Another integrated approach of single-molecule genomic data, spatial agent-based modeling, and statistical inference was recently introduced to derive tumor ancestral trees in patient-specific colorectal cancer samples that lead to the observation of a CSC fraction of 0.5–4% (Sottoriva et al., 2013).

**Table 1:** **Cancer stem cells in solid tumors**.

Tumor type	Cells expressing CSC marker (%)	Minimal number of cells expressing marker for tumor formation	Calculated cancer stem cell ratio
**Breast**	11–35	200	1.1 × 10^−3^
	ND	2000	
	3–10	500	
**Brain**	19–29	100	3.3 × 10^−4^
	6–21	100	
**Colon**	1.8–25	200	5.4 × 10^−4^
	0.7–6	3000	
	0.03–38	200	
**Head and neck**	0.1–42	5000	4.2 × 10^−5^
**Pancreas**	0.2–0.8	100	3.5 × 10^−5^
	1–3	500	
**Lung**	0.32–22	10,000	1.1 × 10^−4^
**Liver**	0.03–6	5000	6 × 10^−6^

*Adapted from Visvader and Lindeman (2008)*.

One mechanism responsible for establishing the CSC fraction within a tumor is the relative frequency with which CSCs either create another CSC (by symmetric division) or a non-CSCs (by asymmetric division) (Caussinus and Hirth, 2007; Dingli et al., 2007b). Mechanisms known to directly affect the symmetric division probability, in turn, include availability of certain host growth factors such as EGF, and growth-factor-rich niches, which can skew division modes in favor of symmetric production of CSC up to 85% (Lathia et al., 2011). Another mechanism responsible for the observed CSC fraction in tumors is factor-independent, and may be traced to the aggregate population-level action of cell proliferation, migration, and apoptosis; a process we have previously described as “self-metastatic” growth (Norton, 2005; Enderling et al., 2009b). Underlying this notion, each CSC can only form a cluster of limited size (Prehn, 1991), until such time as it can opportunistically migrate out of its current cluster to seed a new cluster nearby.

To show how these influences comprising the CSC hypothesis can give rise to realistic tumor growth dynamics and morphologies, we used an agent-based cellular automaton model of tumor population dynamics that considers the kinetics and interactions of CSC and CCs. Consistent with observation, we show that host-dependent variations in (a)symmetric CSC division ratios can yield tumors with substantially different CSC pool sizes and overall tumor morphologies. Furthermore, we show, by virtue of the properties of CSCs and their progeny, that the CSC fraction in a tumor grows over time, regardless of how quickly the tumor as a whole grows. As will be argued, these findings can explain the large variation in CSC fractions within and among tumors reported throughout the literature (Reya et al., 2001; Visvader and Lindeman, 2008), and offer a new paradigm for cancer development within the CSC hypothesis.

## Materials and Methods

We use an agent-based cellular automaton model to describe the behavior of individual tumor cells dependent on intrinsic mechanisms of proliferation, migration, and cell death. By tracking the fate of multiple cells over time we simulated the emergence of interacting tumor cell populations that compete for the same environment (see Deutsch and Dormann, 2005 for an overview of similar approaches). Such theoretical frameworks are increasingly utilized to investigate different aspects of the CSC hypothesis (Deasy et al., 2003; Dingli and Michor, 2006; Ganguly and Puri, 2006; Ashkenazi et al., 2007; Bankhead et al., 2007; Dingli et al., 2007b; Michor, 2008; Piotrowska et al., 2008; Galle et al., 2009; Glauche et al., 2009; Johnston et al., 2010; Sottoriva et al., 2010). Ganguly and Puri (2006) developed a compartment model of normal stem cells, early and late progenitors, and mature cells in the neural lineage as well as their abnormal counterparts. Through numerical simulation of physiologic homeostasis in their deterministic ordinary differential equation model, the authors explored the impact of mutations in the stem cell and early progenitor populations. As stem cells have a larger proliferation potential, they found that mutations in stem cells lead to a larger tumor population growth rate. Considering normal and abnormal stem and differentiated cell populations of the hematopoietic system, Dingli and Michor (2006) showed in a simple ordinary differential equation model that successful therapy must eradicate the CSCs. Therapy that targets mature CCs or partially induces differentiation of stem cells is unable to provide tumor control. Recent mathematical investigations into the fraction of CSCs in solid tumors either assumed a fixed proportion of these cells and explored the cell kinetic parameters in a hierarchical ordinary differential equation model that lead to proportional stability in the cancer lineage (Molina-Peña and Álvarez, 2012), or simulate the exponential phase of tumor growth and observe a constant minor proportion of CSCs in an ordinary differential equation model (Johnston et al., 2010) or in a cellular automaton approach (Morton et al., 2011). Recently, Hillen et al. (2013) used reaction-birth processes and developed a mean-field integro-differential equation system to describe spatio-temporal tumor growth under the CSC hypothesis. Analyzing the simplified ordinary differential equation system of their model, the authors were able to show that tumor growth accelerates with increased cell death and that the tumor population monotonically evolves to a pure CSC state. Using a partial differential equation approach to simulate the spatio-temporal dynamics of cell lineage in solid tumors, Youssefpour et al. (2012) were able to observe complex pattering with CSCs being predominantly located in individual clusters at the outer rim of the total population in response to a variety of cellular feedback mechanisms and oxygen tension. Similar to our agent-based model investigations into CSC-driven solid tumor growth (Enderling et al., 2009a,b), Sottoriva et al. (2010) recently developed a hybrid cellular automaton model to study tumor morphology and phenotypical heterogeneity in the classical cancer model where all cells can be considered CSCs, and in the CSC model with populations heterogeneous for proliferation potential. In addition to increasing invasiveness with decreased CSC fraction, the model revealed a constant CSC fraction during exponential growth phases. While Sottoriva and co-workers assumed intratumoral proliferation through pushing adjacent cells toward the tumor periphery, we set out to explore tumor growth dynamics and morphology evolution when proliferation is restricted to the tumor outer rim as a result of competition for space in the tumor interior (Brú et al., 2003; Drasdo and Höhme, 2005; Galle et al., 2009). We will explore global cancer features in an agent-based model as a function of environmentally modulated CSC symmetric division rates as well as the proliferative potential of the CC population, and discuss evolution of the tumor population and CSC fraction during local tissue invasion.

**Figure 1:**
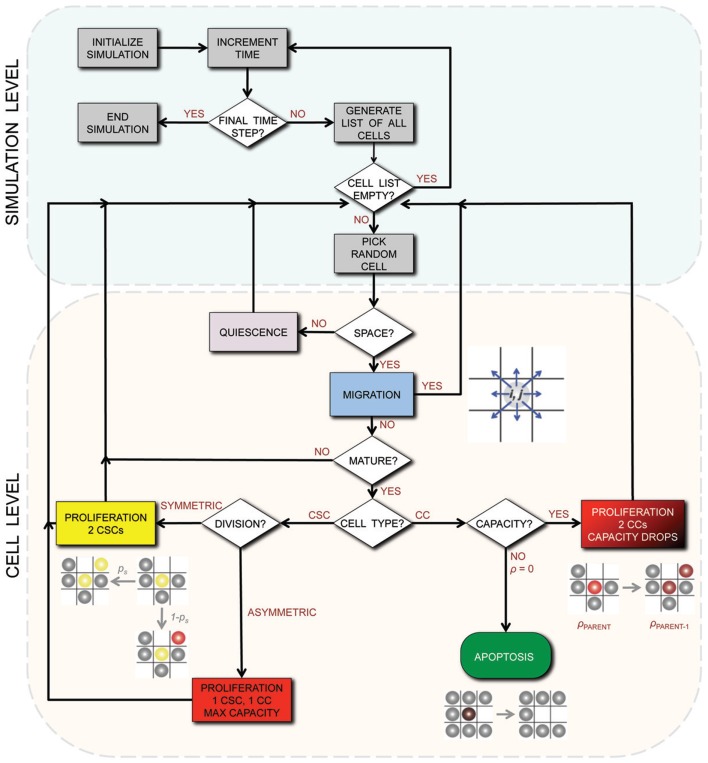
**Flowchart of the simulation process and decisions on the cell level**. CSC, Cancer stem cell; CC, non-stem cancer cell; ρ, proliferation capacity.

A detailed description of the agent-based model assumptions and biological motivation can be found elsewhere (Enderling et al., 2009a,b; Enderling and Hahnfeldt, 2011). To summarize, at time *t* = 0, we initiate a single cell in the center of our computational lattice (domain) of 10 mm × 10 mm, subdivided into 1000 × 1000 equal-sized lattice points of (10 μm)^2^ that can hold at the most one cell at any time. By simulating (a)symmetric cell proliferation, migration, and cell death kinetics at discrete time intervals Δ*t*, a population of cancer cells emerges, which we track until it reaches 100,000 cells. In this procedure, we assume CCs are able to proliferate a certain number of times, ρ_max_, before inevitable cell death and ultimate removal from the simulation. For CSCs, we assume ρ_max_ = ∞. CSCs divide symmetrically with fixed probability *p*_s_, and asymmetrically with probability 1 − *p*_s_. Cells need to mature through the cell cycle before division can occur, which takes a cell-type-dependent time τ. With available adjacent space, a cell can migrate with rate μ. Otherwise, the cell is forced into quiescence until space becomes available. With probability α, CCs undergo spontaneous cell death and vacate the space they occupy. CSCs are assumed to be immortal (α = 0). A flowchart of the simulation process and decisions at the cell level is shown in Figure 1.

## Results

### Symmetric stem cell division probability *p_s_* and tumor growth

Tumor growth is simulated and analyzed for various stem cell division probabilities *p*_s_ = (0.1, 0.25, 0.5, 0.75, 0.99) and various progeny proliferation capacities ρ_max_ = (10, 15, 20), which are in line with reported progeny division potentials for different tissues (Bernard et al., 2003; Ashkenazi et al., 2007). As previously shown, tumors in which CCs have high values of ρ_max_ grow significantly slower than tumors with CCs with limited proliferation capacity (low ρ_max_) (Enderling et al., 2009a). With proliferation being dependent on available space, tumor growth is restricted to cells on the outer rim of cell clusters, which is frequently observed *in vitro* and *in vivo* (Brú et al., 2003; Drasdo and Höhme, 2005; Galle et al., 2009). CSCs become “trapped” in the tumor core and are forced into quiescence until space becomes available again – either after adjacent CCs have migrated away or died. These kinetics have been shown to be inversely dependent on CC proliferation capacity and death rate (Enderling and Hahnfeldt, 2011; Morton et al., 2011).

**Figure 2:**
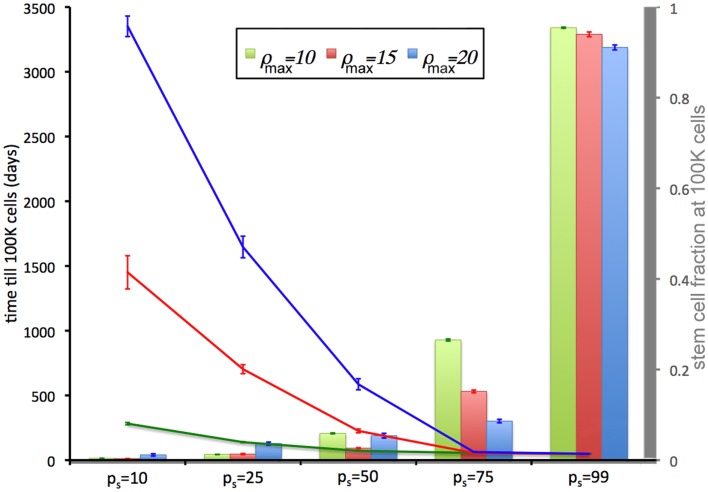
**Tumor development time and stem cell fractions depend on progeny proliferation capacity ρ_max_ and stem cell symmetric division probability *p*_s_**. The time until the tumor reaches 100,000 cells (lines) decreases with increasing *p*_s_. For small *p*_s_ (*p*_s_ = 10%, 25%), the stem cell fraction (columns) increases with increasing progeny proliferation capacity ρ, but for larger *p*_s_ the trend reverses. For *p*_s_ = 50% there is a *U*-shape dependence on ρ_max_. For *p*_s_ ≤ 75%, the stem cell fraction is substantially less than the symmetric division probability *p*_s_. For *p*_s_ = 50%, the average stem cell fraction in a 100,000-cell tumor is less than 5% for each ρ_max_.

We now relate the effect of symmetric CSC division probability *p*_s_ on tumor growth. A high symmetric CSC division probability (*p*_s_ = 0.99; i.e., 99%) is found to yield tumors with a very large CSC population (93%). Tumors of 100,000 cells are formed within 49 days regardless of the CC proliferation capacity. Lower symmetric stem cell division probabilities of *p*_s_ = 75% and *p*_s_ = 50% result in both a smaller stem cell compartment (15.5 and 5.4%, respectively) and more progeny CCs that act to encapsulate and slow the expansion of the available CSCs, prolonging tumor growth to 56 and 72 days, respectively, for ρ_max_ = 10, and 56(63) and 226(586) days for ρ_max_ = 15(20) (Figure 2). CSC fractions are reduced further to 1.8 and 0.5% for *p*_s_ = 25% and 10%, respectively. In the latter case, tumor growth takes as long as 3351(1451 and 282) days for ρ_max_ = 20(15 and 10), respectively.

The intrinsic and/or extrinsic mechanisms that regulate (a)symmetric CSC division are not yet understood, but modeling predicts that if the symmetric division probability is even modestly less than 100% – which it must be given the observed cell fate heterogeneity within a tumor – the CSC compartment rapidly becomes a minor subpopulation within tumors (Figure 2) and remains that way at least for a long while.

### Symmetric stem cell division probability *p_s_* and tumor morphology

**Figure 3:**
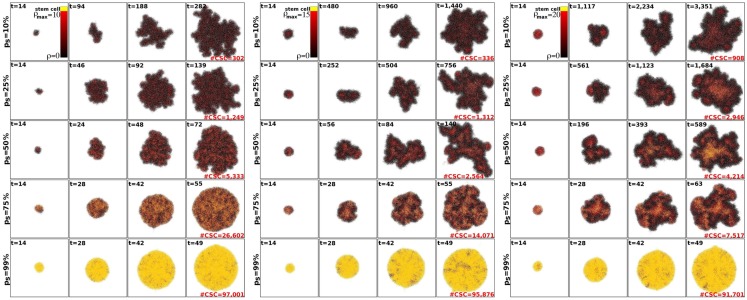
**Representative tumor morphologies for symmetric division probabilities *p*_s_ = 10, 25, 50, 75, and 99% (rows), and for ρ_max_ = 10, 15, and 20 (panels left to right)**. All tumors grown to 100,000 cells.

Within the CSC hypothesis tumor growth follows an interesting pattern. CSCs can only form tumor clusters of limited size before self-inhibition (Prehn, 1991). Tumor growth and progression is achieved by shedding of CSCs from the tumor clusters and seeding of new clusters nearby – a process termed self-metastatic growth (Norton, 2005; Enderling et al., 2009b, 2010). Figure 3 shows the spatio-temporal evolution of tumor morphology for various symmetric stem cell division probabilities and thus various stem cell pool sizes within the tumors. As expected, and in line with the literature, homogeneous tumor populations consisting of only stem cells (or a sufficiently large stem cell compartment) grow in a radially symmetric manner (Drasdo and Höhme, 2005; Enderling and Hahnfeldt, 2011). Small symmetric stem cell division probabilities, by contrast, result in CSCs being scattered throughout the tumors through self-metastatic progression. As this continues, the tumor clusters become bigger and intermingle, giving rise to clusters of stem cells, likened to stem cell niches in normal tissues (Figure 3).

**Figure 4:**
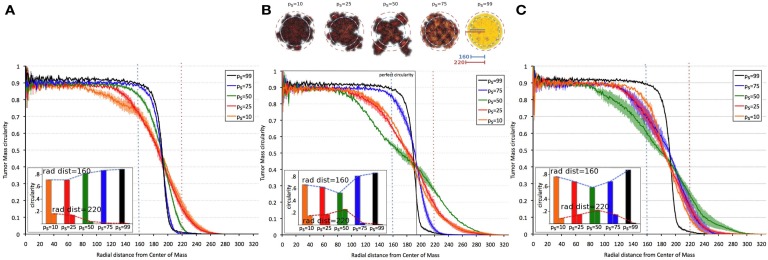
**Dependence of tumor morphology on stem cell symmetric division probability and proliferation capacity ρ_max_ = 10 (A), 15 (B), 20 (C)**. Plotted against radial distance from the center of mass of the tumor is tumor “circularity” (the percent occupancy of lattice points by tumor cells at the given radial distance), a measure of the tumor’s irregularity/invasiveness. For ρ_max_ = (15, 20), the dependence of circularity on *p*_s_ is *U*-shaped. All tumors grown to 100,000 cells.

Tumor morphology is often used as a measure of tumor invasiveness. A compact, circular morphology is associated with less invasiveness than an irregular, fingering morphology (Anderson, 2005; Anderson et al., 2006). To assess this, we measured tumor compactness, or “circularity,” i.e., tumor density as a function of radial distance from the tumor’s center of mass, for various symmetric division probabilities *p*_s_ (Figure 4). It is observed that tumor morphology can be described by three distinct regions: (i) a non-linear (saturated) region without significant changes over time in the bulk tumor core close to the center of mass, (ii) a deterministic region dominated by cell proliferation and diffusion, and (iii) a highly fluctuating outer rim zone distant from the center of mass with a low cell density that is dominated by random cell migration (Hatzikirou et al., 2010). Tumors comprised primarily of CSCs form a very dense, saturated tumor without a largely fluctuating outer rim, as the effect of random migration at the tumor boundary is nullified by continuous stem cell proliferation. A smaller dense core and a larger deterministic and highly fluctuating self-metastatic region characterize tumors with lower stem cell fractions. Of note, in tumors with larger progeny proliferation capacities [ρ_max_ = (15, 20)] there is a non-linear, *U*-shaped dependence of tumor compactness on stem cell fraction. Smaller and larger symmetric stem cell division probabilities *p*_s_ yield a more compact tumor morphology, whereas a symmetric division probability of *p*_s_ = 50% features the most invasive morphology with the smallest dense core and the most widespread proliferative and motile regions.

### Stem cell fraction as a function of time

**Figure 5:**
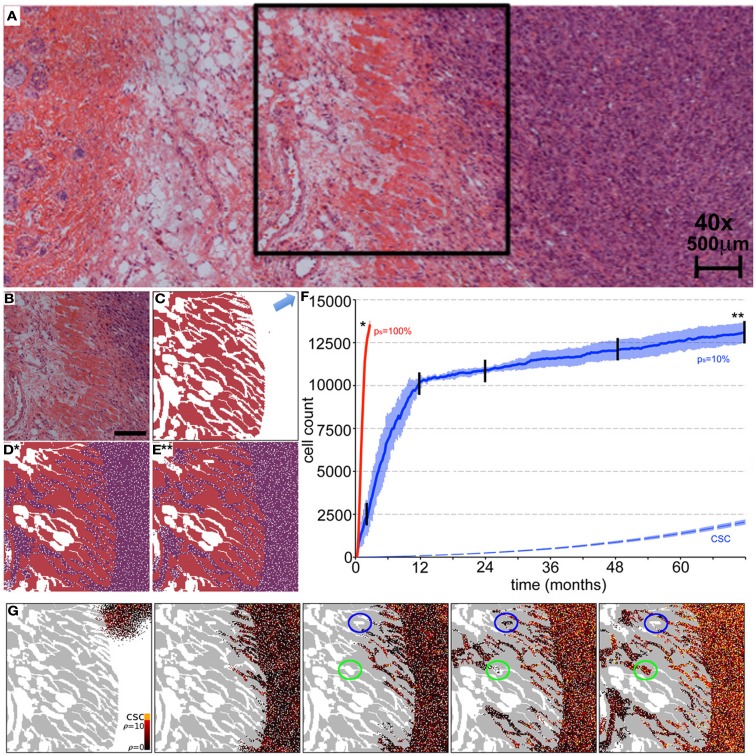
**Representative simulations of tumor growth from a single cancer stem cell and invasion of adjacent muscle and fat tissue**. **(A)** H&E staining of an LLC tumor – host tissue interface. **(B)** Close-up of the marked region in **(A)** of the tumor outside and invading the muscle/fat tissue. Scale bar = 500 μm. **(C)** Initial condition for the computer model. Domain is initialized using an image mask of the muscle tissue identified in **(B)**. A single cancer stem cell is placed in the top right corner (arrow). **(D,E)** *In vivo* tumor morphology is dictated by host tissue architecture regardless of intrinsic tumor kinetics. **(D)** Cancer cells (purple) in a pure stem cell tumor (*p*_s_ = 100%) have proliferated within the space and invaded the muscle. The simulation snapshot correlates to the time point marked by * in **(F)**. **(E)** Same as Case **(D)** but for the heterogeneous tumor (*p*_s_ = 10%). The simulation snapshot correlates to the time point marked by ** in **(F)**. **(F)** Number of tumor cells over time for symmetric stem cell division probabilities *p*_s_ = 100% (red line) and *p*_s_ = 10% (blue plot). The simulation is run until both tumors reach a comparable size of 15,000 cells. The tumor composed purely of stem cells reaches this size after 100 days, whereas the heterogeneous tumor (*p*_s_ = 10%) takes more than 72 months. In the latter case, the area outside the muscle is completely occupied after 12 months, harboring 60 cancer stem cells (dashed blue plot). By time 72 months, the number of cancer stem cells outside the muscle has increased to 2050. Shown are the averages and standard deviations of 10 independent simulations **(G)** Representative simulation snapshots of different time points of tumor growth and invasion in a tumor with *p*_s_ = 10%. The time points are marked in **(F)** with black vertical lines. The space adjacent to the muscle is quickly occupied, whereas the invasion of the muscle architecture takes a long time. Stem cells must invade to seed new cells in the less dense tissue within and beyond the muscles, but their invasion is inhibited by their non-stem offspring with limited proliferation capacity. Non-stem microtumors cannot be sustained and disappear over time (blue circles). Microtumors seeded by a stem cell manifest and become very stemmy (green circles). The stem cell pool in the tumor adjacent to the muscle increases steadily over time as non-stem daughter cells die off over time (α = 1%).

**Figure 6:**
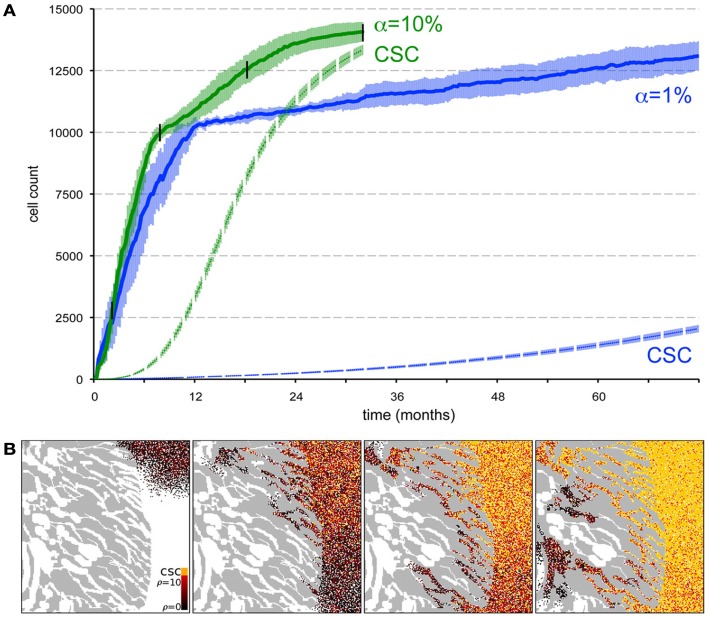
**Spontaneous cell death yields a higher stem cell fraction within the tumor and promotes tumor progression**. **(A)** Comparison of tumor growth curves for tumors with spontaneous cell death rates α = 1% (blue plot) and α = 10% (green plot). Other parameters *p*_s_ = 10% and ρ_max_ = 10. The dashed plots show the number of cancer stem in the tumor population adjacent to the muscle for both tumors. Shown are the averages and standard deviations of 10 independent simulations. **(B)** Snapshots of different time points of tumor growth and invasion in a tumor with α = 10%. The time points are marked in **(A)** with black vertical lines. The stem cell pool in the tumor adjacent to the muscle increases rapidly over time as non-stem daughter cells die off quickly compared to α = 1% in Figure 5G.

Beyond the dependence of tumor growth dynamics on cell kinetic parameters, environmental constraints may also modulate tumor progression and architecture. Tumors growing *in vivo* compete with the environment not only for oxygen and nutrients (which are not very limiting in the early phases of tumor growth), but also for space to grow and proliferate. Different host tissues, such as epithelial membranes or dense muscle structures (Figure 5) retard tumor progression. Single cells will eventually be able to infiltrate the tissue and thus form a path for local invasion. We introduce a “host tissue” in our computational domain by discretizing a hematoxylin-eosin (H&E)-stained tissue sample of a murine Lewis lung carcinoma – mouse muscle tissue interface. For the purpose of our study, we did not model anatomically precise cell structures but limited the tissue structure to average local cell densities (Figure 5). We introduce a single CSC in the top right corner of the domain and simulate tumor growth with either *p*_s_ = 100%, representing a pure CSC tumor, or *p*_s_ = 10%. Tumors consisting solely of stem cells quickly populate the empty space representative of tissue with low density, and with a reduced growth rate into the dense muscle structure and less dense tissue areas beyond. Heterogeneous tumor populations (*p*_s_ = 10%) populate the less dense tissue via formation of self-metastases, as previously described (Enderling et al., 2009b), and the denser structures with a further reduced growth rate. Snapshots of the spatial distribution of 13,000 tumor cells for both tumors (*p*_s_ = 100 and 10%) reveal no difference in morphology (Figure 5). The circular and self-metastatic tumor morphologies evident in the respective tumors without host constraints (c.f., Figure 3) are no longer present. Instead, *in vivo* tumor morphology is dictated by host tissue architecture. What is seen, however, is a significant difference in tumor growth and growth rate history. While stem cell tumors fill the available space and form a mass of 13,000 cells within about 100 days, the heterogeneous tumor takes 20 times longer (72 months) to reach a comparable size and morphology (Figure 5). In the presented sample simulation, the empty space to the right of the muscle tissue is populated by the heterogeneous tumor within 12 months, but the invasion of the muscle structure takes significantly longer (another 60 months), during which time the overall CSC fraction goes from 60/9500 (0.6%) to 2050/13,000 (15.8%). Simulation snapshots at different time points clarify the heterogeneous tumor growth dynamics and stem cell fraction evolution (Figure 5). When the tumor population reaches the dense muscle structure, single cells try to invade the narrow gaps between muscle cells. As more than 99% of the tumor is comprised of CCs at the time invasion commences, the infiltrating and invading cells can only form small clusters of cells that eventually die out, blocking invasion routes, and inhibiting tumor invasion. Only the eventual opportunistic infiltration of a CSC results in successful metastatic seeding of these interstices within the muscle architecture. Figure 5 shows how these spaces fill with microscopic cancer nodules that over time disappear again, awaiting the chance entry of a stem cell. The necessity of rare CSCs, which are predominantly migration- and proliferation-inhibited by their own progeny as well as by host tissue, to infiltrate and invade host tissue structures can, at least in part, explain the frequently observed poor efficiency of metastization (Luzzi et al., 1998; Wyckoff et al., 2000; Dingli et al., 2007a). As remarked, the overall CSC frequency increases over time as dying CCs get opportunistically replaced by CSCs. Such a time-dependent tumor stem cell fraction offers one plausible explanation for the wide spread of stem cell fractions reported in the literature even for tumors of the same tissue of origin (Quintana et al., 2008; Visvader and Lindeman, 2008) (c.f., Table 1). These results are amplified when the CC death rate is higher. With competition for space being a pivotal tumor-inhibiting factor, the introduction of a 10-fold higher spontaneous death rate α in CCs results in accelerated tumor growth and host tissue invasion, along with a more rapid increase in CSC number and percentage within the tumor (Figure 6).

## Discussion

The existence of a minor subpopulation of CSCs within a tumor that drives tumor initiation, growth, and progression is an attractive hypothesis to explain primary tumor dynamics and transplantation experiments. The existence of a minor fraction of CSCs in leukemia has long been appreciated (Furth and Kahn, 1937), but over the last 10 years or so, CSC-like populations have also been reported in solid tumors of, for example, the breast, brain, prostate, and colon (Al-Hajj et al., 2003; Singh et al., 2003; Cammareri et al., 2008; Hurt et al., 2008). The reported frequencies with which these cells occur in a tumor varies by many orders of magnitude; dependent, for example, on the chosen experimental setup and purification methods (Quintana et al., 2008; Visvader and Lindeman, 2008). Furthermore, the size of the CSC pool can be modulated by availability of certain host growth factors like EGF (Lathia et al., 2011), Sonic hedgehog (Takezaki et al., 2011), Wnt (Vermeulen et al., 2010), or Notch (Wang et al., 2009), supporting the idea of a CSC niche (He et al., 2009; Borovski et al., 2011). Here we present a cellular automaton model of tumor growth and invasion of heterogeneous cancer populations comprised of CSCs and their progeny. Simulations of the model reveal multiple indications on the fraction of CSCs in solid tumors: (i) intrinsic stem cell symmetric division probabilities results in tumors with different stem cell ratios and morphologies, (ii) CSCs are intrinsically a minor subpopulation within a tumor, and (iii) the stem cell ratio within a tumor is variable over time with intratumoral and environmental competition for limited resources – space in the case of muscle invasion – selecting for and thus enriching in CSC fraction. In fact, competition such as for space and the resulting selection for CSCs has recently been shown to yield a pure CSC population over time (Hillen et al., 2013).

The frequency of symmetric division events in CSCs has previously been identified as a pivotal determinant of stem cell proportion experimentally (Cicalese et al., 2009) as well as in a variety of theoretical approaches including differential equations (Johnston et al., 2010), agent-based approaches (Enderling et al., 2009a), and hybrid models (Sottoriva et al., 2010).

CC proliferation capacity and the space- and time-dependent evolution of the CSC fraction in solid tumors offers a novel augmentation to the ongoing discussion about the frequencies at which CSCs are observed (Pardal et al., 2003; Quintana et al., 2008; Visvader and Lindeman, 2008). Furthermore, the CSC fraction, as determined by host environmental factors that control the symmetric division probability, determines whether the tumor exhibits an invasive or compact morphology, although the relationship is non-monotonic. Intrinsic tumor growth can be described as conglomerates of self-metastases (Enderling et al., 2009b, 2010), but over time a solid tumor core forms with more or less invasive boundary clustering. While lower and higher stem cell fractions yield more compact morphologies, intermediate fractions result in the most invasive tumor morphologies. These results augment findings of a monotonic increase of invasiveness with decreased stem cell fraction (Sottoriva et al., 2010). When additional host spatial constraints are imposed on the growing tumor, intrinsic morphological features disappear. Conceivably, the indistinguishable pathological morphologies that result could, in a clinical setting based on empirical observations, lead to the recommendation of comparable treatment protocols. However, due to their different CSC fractions, morphologically comparable tumors could in fact demonstrate response patterns ranging from complete regression [for low CSC content (Enderling et al., 2009c)] to resistance and accelerated re-growth (Gao et al., 2013).

Herein we limited our study to early avascular tumor growth where the total population is sufficiently small such that oxygen diffusion and tension can be neglected and global tumor growth dynamics be derived from different intrinsic CC kinetics. Simulations of larger tumor volumes will require physiological extension of the model to include nutrient delivery (Anderson and Chaplain, 1998; Ribba et al., 2004; Frieboes et al., 2007; Macklin et al., 2009) and vascular carrying capacities (Folkman, 1971; Folkman and Hochberg, 1973; Hahnfeldt et al., 1999). Furthermore, a translation from the cellular level model to a tissue-level continuous description might be more feasible to augment our understanding of the dynamics of larger populations (Hillen et al., 2013). For computational convenience we also limited this study to two spatial dimensions, but emphasize that extension to three spatial dimensions is algorithmically straightforward (Enderling et al., 2009b), with no qualitative change to be expected in the results here reported.

However, one transcendent feature expected to survive model simplifications is the possibility of widely varying stem cell compositions, highly dependent on host structural and biochemical context. This finding needs to be taken into account in both the clinical and research arenas, where heretofore, the threat has been presumed to come from the tumor bulk as a whole, not from a limited subpopulation within it.

## Conflict of Interest Statement

The authors declare that the research was conducted in the absence of any commercial or financial relationships that could be construed as a potential conflict of interest.
